# Validation of a brief mental health screening tool for pregnant women in a low socio-economic setting

**DOI:** 10.1186/s40359-019-0355-3

**Published:** 2019-12-09

**Authors:** Zulfa Abrahams, Marguerite Schneider, Sally Field, Simone Honikman

**Affiliations:** 1 Perinatal Mental Health Project Alan J Flisher Centre for Public Mental Health, Department of Psychiatry and Mental Health, University of Cape Town, The Annex, 46 Sawkins Road, Rondebosch, Cape Town, 7700 South Africa; 2 Alan J Flisher Centre for Public Mental Health, Department of Psychiatry and Mental Health, University of Cape Town, Cape Town, South Africa

**Keywords:** Common mental disorders, ROC analysis, Cognitive testing, Low-resource setting, Pregnancy

## Abstract

**Background:**

In South Africa, the prevalence of symptoms of common mental disorders (CMD), i.e. depression, anxiety and suicidal thoughts are high. This study aimed to use a cognitive interviewing technique to validate the content and structure of a 4-item screening tool, to adapt the tool accordingly, and to use receiver operating curve (ROC) analysis to determine the optimum cut-point for identifying pregnant women with symptoms of CMD.

**Methods:**

We conducted a mixed method study at a Midwife Obstetric Unit in Cape Town. Women attending the clinic for their first antenatal visit during the recruitment period, whose first language was English, Afrikaans or isiXhosa, were invited to participate. A 4-item screening tool was administered in the first language of the interviewee, after which a cognitive interviewing technique was used to examine the question-response processes and considerations used by respondents as they formed answers to the screening tool questions. The Edinburgh Postnatal Depression Scale (EPDS) was used to identify women with symptoms of CMD.

**Results:**

A 2-week recall period performed well. Questions about (1) being unable to stop worrying, or thinking too much, (2) feeling down, depressed or hopeless, and (3) having thoughts and plans to harm yourself, were well understood. The question that referred to feeling little interest or pleasure in doing things, was poorly understood across all languages. Using ROC analysis with the EPDS as the reference standard, and a cut-point of ≥13, we showed that a 3-item version of the screening tool was able to correctly classify 91% of the women screened.

**Conclusions:**

Cognitive interviewing enabled testing and refining of the language and constructs of an ultra-brief screening tool. The shortened, 3-item tool is well understood and effective at identifying pregnant women with symptoms of CMD, across the three most commonly spoken languages and cultures in Cape Town.

## Background

In developed countries, the prevalence of maternal depression ranges between 7 and 15% [1], while in low- and middle-income countries (LMIC), the prevalence measured by both screening or diagnostic tools are as high as 20–26% [2]. In addition to depression, evidence suggests that anxiety occurs frequently during pregnancy, and may be even more common than depression. In a systematic review of anxiety during pregnancy, Brunton et al. reported global prevalence rates ranging from as low as 18% to as high as 60% [3]. Suicidal ideation and behaviour have also become increasingly reported during the perinatal period, with prevalence rates of between 6 and 18% [4–7]. In South Africa, similar to many other LMIC, the prevalence of depression, anxiety and suicidality is high. A recent study in Cape Town reported that the diagnostic prevalence of maternal depression was 22% [8], anxiety was 23% [9] and suicidal ideation and behaviour was 18% [4].

Common mental disorders (CMD), defined as symptoms of depression and anxiety, are of particular concern during the perinatal period because of its disabling effect on maternal functioning and on social and economic self-fulfilment, as well as the negative consequences for the health and development of infants and children [10]. Globally, about 80% of women affected by CMD during the perinatal period are not identified or treated [11]. At the time this research was conducted, routine screening for symptoms of CMD was not provided in South African primary care antenatal settings, despite the South African Mental Health Act explicitly stating that mental health care should routinely be provided within the general health environment, at primary and community level. The absence of routine screening was partly due to the lack of a short, simple and easily administered screening tool.

Both the Whooley questions and the Edinburgh Postnatal Depression Scale (EPDS) [12–15] have been validated against diagnostic criteria in research contexts in South Africa. In Johannesburg, the EPDS was validated against the Diagnostic and Statistical Manual (DSM-IV) [16] criteria for depression in a sample of postnatal women [12]. The study found that when using a threshold of 11, the EPDS identified 100% of women with major depression and 70.6% of women with minor depression (sensitivity = 80%; specificity = 76.6%). In a study in Cape Town, the anxiety subscale of the EPDS - which consists of questions 3, 4 and 5 - was validated against the Mini-International Neuropsychiatric Interview diagnostic criteria [17] and found to correctly classify 61% of the sample of pregnant women (Area Under the Curve (AUC) = 0.69; sensitivity = 67%; specificity = 59%) [15].

Even though the EPDS has been validated in research settings in South Africa, its structure is not feasible to be routinely used in busy, low resource primary care settings by non-specialist health workers due to its length (10-items) and Likert scoring system. Furthermore, several of the idiomatic constructs embedded in this Scottish-derived tool are culture-bound, e.g. “things have been getting on top of me” and “seeing the funny side of things”. These idioms are poorly understood in the typical South African linguistic context, unless careful explanations are given, such as can occur in research settings. Aside from screening administrators themselves potentially misunderstanding the items, it is time-consuming to explain the meanings of poorly understood items and thus, this would not logistically be feasible in the typical service environment. The Whooley questions, which consist of two items, with a possible third item, have also been validated in South Africa, [15] but generalisability of the results of this study is limited as a psychiatrist conducted both the screening and diagnostic procedures.

To address the gap between the too long EPDS and too short Whooley questions, the Perinatal Mental Health Project (PMHP) developed an English language, 4-item screening tool, for identifying pregnant women with symptoms of CMD and suicidal ideation in a low socio-economic setting in South Africa [18]. In the tool’s development, psychometric analysis was used to compare the performance of several commonly used screening tools, and the individual items within these tools, against the reference standard performance of the Expanded MINI (MINI Plus Version 5.0.0) clinical diagnostic interview [17]. Using Receiver Operating Characteristic (ROC) analysis with the MINI as the reference standard, this 4-item tool correctly classified 75% of the sample of women, when a cut-point of two out of a possible four was used (AUC = 0.76; sensitivity = 65%; specificity = 82%) [18]. The 4-item screening tool (Table 1) was derived from the Whooley [19], the Generalised Anxiety Scale (GAD-2) [20] and the EPDS [13]. The GAD and EPDS items were converted from their original Likert format to binary format for consistency and the time recall period was standardised to the prior 4 weeks.

**Table 1:** Questions making up the screening tool used in Phase 1 and Phase 2 of validation process

Phase 1	Origin
In the last 4 weeks, have you often (on some or on most days)	
1. Felt unable to stop worrying, or thinking too much?	GAD-2
2. Felt down, depressed or hopeless?	Whooley-1
3. Felt little interest or pleasure in doing things that you used to enjoy before?	Whooley-2
4. Had thoughts and plans to harm yourself or commit suicide?	EPDS-10
Phase 2	
In the last 2 weeks, have you on some or most days	
1. Felt unable to stop worrying, or thinking too much?	GAD-2
2. Felt down, depressed or hopeless?	Whooley-1
3. Been concerned/troubled about having little interest or pleasure in doing things?	Whooley-2
4. Had thoughts and plans to harm yourself or commit suicide?	EPDS-10

This study aims to further validate the 4-item screening tool [18] by (1) using a cognitive interviewing technique, in three local languages, in a sample of pregnant women from a low resource setting, to validate the content and structure, i.e. the construct validity of the 4-item screening tool, (2) adapting the tool accordingly, and (3) using ROC analysis, with the EPDS as the reference standard, to determine the optimum cut-point to be used to identify symptoms of CMD in pregnant women.

## Methods

This study used a mixed method design and was conducted in Cape Town, South Africa between September and October 2017. Data were collected using questionnaires (quantitative) and semi-structured interviews (qualitative).

An amendment to the initial PMHP study’s ethical approval was obtained from the Human Research and Ethics Committee at the University of Cape Town (HREC REF: 131/2009). The Western Cape Provincial Department of Health approved the use of the research site. Participants who were identified as needing mental health support were referred to a qualified, on-site counsellor for free services. All participants were informed that they were free to withdraw from the study at any time without consequences. Those who participated in the study provided written, informed consent (and unassisted consent in the case of participants younger than 18 years, as the study was linked to a therapeutic intervention that did not require parental consent) after the procedure had been verbally explained to them. Consent forms were available in English, Afrikaans and isiXhosa. No financial incentives were provided for participating in the study.

### Setting

This study was conducted at the Hanover Park Midwife Obstetric Unit (MOU), a public, primary healthcare facility in Cape Town, South Africa. The Hanover Park MOU offers free antenatal and postnatal services to pregnant and postpartum women. Approximately 10–15 women attend this facility for their first antenatal appointment every day. Hanover Park is a low-income, residential suburb that experiences high rates of gang activity, violent crimes and school drop-out [21]. More than half the women attending the MOU are unemployed, while 42% are considered to be food insecure [22].

### Participants

Women attending the MOU for their first antenatal visit during the recruitment period, whose first language was English, Afrikaans or isiXhosa, were invited to participate in the study. As home language is related to race, income and education in the South African context, due to the legacy of Apartheid [23], the demographic profile of the participants are presented in Table 2. A total of 66 women, aged between 15 and 38 years (mean = 27.5; SD = 5.7), consented to being interviewed and having their interview recorded in a private interview room. None of the women who were invited to participate, declined. The most commonly spoken home language was English (*n* = 30; 45.4%), followed by Afrikaans (*n* = 19; 28.8%) and IsiXhosa (*n* = 17; 25.8%). Women who spoke isiXhosa were significantly older than those who spoke English or Afrikaans (*p* = 0.035). Women who spoke Afrikaans had significantly more pregnancies (*p* = 0.031) and a lower level of education than women who spoke English or isiXhosa (*p* = 0.020). The prevalence of depression (defined as scoring < 13 on the EPDS) was 24.3% (*n* = 16). Significantly fewer women who spoke English (*n* = 3; *p* = 0.042) screened positive for depression using the EPDS, compared to those who spoke Afrikaans (*n* = 7) and those who spoke isiXhosa (*n* = 6). The majority of women were in the first trimester of their pregnancy.

**Table 2:** Demographic characteristics of women by language of interview

	English (*n* = 30) Median (IQR)	Afrikaans (*n* = 19) Median (IQR)	isiXhosa (*n* = 17) Median (IQR)	*P*-value*
Age	26.1 (23.8–29.3)	26.0 (21.5–33.7)	31.0 (28.0–35.9)	0.035
Pregnancies	2 (1–3)	3 (2–3)	2 (2–3)	0.031
Live births	1 (0–2)	1 (0–2)	1 (1–2)	0.382
	n (%)	n (%)	n (%)	*P*-value**
Highest grade completed				
Grade 7–9	6 (20.0)	9 (47.4)	0	0.020
Grade 10–12	22 (73.3)	10 (52.6)	17 (100)	
Diploma	2 (6.7)	0	0	
Employment status				
Employed	12 (40.0)	4 (21.1)	8 (47.1)	0.244
Unemployed	16 (53.3)	15 (79.0)	9 (52.9)	
Student	2 (6.7)	0	0	
Edinburgh Postnatal Depression Scale				
Negative (score < 13)	27 (90.0)	12 (63.2)	11 (64.7)	0.042
Positive (score ≥ 13)	3 (10.0)	7 (36.8)	6 (35.3)	

*Kruskal Wallis test

**Fisher’s exact test

### Data collection

Questionnaires and a structured interview guide were translated and adapted using the World Health Organisation (WHO) recommended method of forward and back-translation [24]. English questionnaires (EPDS and 4-item screening tool) and a structured interview guide were forward translated into Afrikaans and isiXhosa by health professionals who were bilingual and familiar with the terminology used in the tools. During the translation process, emphasis was placed on the conceptual and cultural equivalence versus the linguistic equivalence of words and phrases. For each of the translated languages, an expert panel convened to identify and resolve the ambiguous expressions and discrepancies between the forward translation and the original English version. The Afrikaans and isiXhosa versions were then back-translated into English by a different health professional who was familiar with the terminology used in the tools, as well as the language nuances of the local community. The Afrikaans and isiXhosa tools were then pre-tested on one individual, representative of each of the target populations. The final version of the tools resulted from two iterations of this process.

First-language English, Afrikaans and isiXhosa speaking women fieldworkers, who had a Bachelor’s degree and professional counselling experience, were trained to seek consent and administer the questionnaires and semi-structured interviews. Pregnant women, attending the MOU for their first antenatal appointment were approached in the waiting areas between routine assessments. The interview process took between 15 and 30 min to complete, was conducted in the participants’ first language, and took place between routine assessments. A socio-demographic questionnaire was used to collect information on participants’ age, number of pregnancies, level of education and employment status. Thereafter, the 4-item screening tool was administered, and responses captured. This was followed immediately by a semi-structured interview which was audio-recorded. The qualitative interview did not affect the responses to the 4-item screening tool.

Cognitive interviewing or question testing is a technique involving a systematic, in-depth approach to assessing the validity of questionnaire content and structure [25–27]. This technique is based on a theory that distinguishes four stages of cognitive processing in response to questioning – understanding, memory, assessment and response [28]. It is used to determine the ways in which participants interpret questions and apply those questions to their own lives, experiences, and perceptions. It is used to investigate how different groups of participants may interpret or process questions differently.

The question-evaluation method of cognitive interviewing [25] was used to examine the question-response processes and considerations used by participants as they formed answers to the screening tool questions. The interview structure consisted of participants providing information to reveal the thinking processes behind their particular answers to the four screening questions. They were asked why they answered the questions as they did, in order to identify problems with interpretive errors and recall accuracy. Interviewers probed participants for concrete examples to support their item responses. Once the interviews were completed, the EPDS was administered. The EPDS, using a cut-point of 13 or more [29, 30], has been found to identify depressive symptoms in South African antenatal women in studies that used diagnostic data as reference standards. The results from both the EPDS and the 4-item screening tool (the latter using a cut-point of ≥2) were used to identify women with symptoms of CMD in the study sample. Women who screened positive on either of the tools were referred to the on-site, mental health counsellor for counselling and support.

A two-phased, iterative approach was used. Phase 1 consisted of interviewing approximately 30 participants, including approximately 10 from each language group. Recruitment continued until data saturation had been reached, i.e. when no new information was discovered in the data analysis. In an attempt to reduce the response error in Phase 1, the interviews were analysed, and adaptations made to the screening tool. One of the adaptations included changing the four-week recall period to 2 weeks, to align with the recall period used in diagnostic interviews. Phase 2 consisted of using the adapted screening tool to interview an additional 36 women (Table 1). Recruitment continued until data saturation had been reached.

### Data analysis

The semi-structured interviews that were conducted in English were transcribed, while interviews conducted in Afrikaans and isiXhosa were translated into English and transcribed by native speakers of each language trained by the lead author. The interview text was analysed separately by two researchers with a third researcher resolving any differences. Analysis included determining how various constructs were understood based on a list of pre-selected criteria developed by all the authors (Table 3).

**Table 3:** Coding of constructs

	In scope interpretations	Examples of in scope interpretations	Out of scope interpretations	Examples of out of scope interpretations
Phase 1				
4-week time recall	Identifies the recall period as 4 weeks	• The last 4 weeks • Within this month • 4 weeks ago	Refers to an event or a time period that is longer or shorter than the 4 weeks	• The past 6 months • Since I found out I was pregnant • The last 2 weeks • The week before the concert • Last week
‘often (on some or most days)’	Expresses the feeling occurring on some or most days	• Everyday • On most days • Most of the time • A lot	Expresses the feeling occurring infrequently/occasionally	• Twice • The weekend • Sometimes
‘felt unable to stop worrying or thinking too much’	Describes the feeling using other phrases or synonyms	• Thinking too much • Cannot stop or relax • Stressed	Describes the feeling using out of scope phrases or synonyms	• Lazy • To be scared
‘felt down, depressed or hopeless’	Describes the feeling using other phrases or synonyms	• Sad • Stressed • Emotional • You just feel stuck	Describes the feeling using out of scope phrases or synonyms	• Aggressive • Frustrated
‘felt little interest or pleasure in doing things you used to enjoy before’	Describes the feeling using other phrases or synonyms	• No lust for life • Feeling hopeless • Moody • Irritable	Describes the feeling using out of scope phrases or synonyms	• Tired • Drained • Lazy
‘had thoughts and plans to harm yourself or commit suicide’	Describes the feeling using other phrases or synonyms	• Kill yourself • Hurt the baby • Don’t want to live anymore	Describes the feeling using out of scope phrases or synonyms or had thoughts but had not made plans	No out of scope interpretations
Phase 2				
2-week time recall	Identifies the recall period as 2 weeks	• This week and last week • In the last 2 weeks • Between 1 and 2 weeks	Refers to an event or a time period that is longer or shorter than the 2 weeks	• 3 weeks ago • The start of my pregnancy – about 5 months ago • In the last week • When my mom was ill
‘on some or most days’	Expresses the feeling occurring on some or most days	• Everyday • A few times per week • Many times	Expresses the feeling occurring infrequently/ occasionally	• Once - on Wednesday • Twice • The weekend • 4 times
‘felt unable to stop worrying or thinking too much’	Describes the feeling using other phrases or synonyms	• To over think • Your mind works overtime • Anxious • Stressed	Describes the feeling using out of scope phrases or synonyms	• Sad • Unhappy
‘felt down, depressed or hopeless’	Describes the feeling using other phrases or synonyms	• Feels like no one cares • Your world is falling apart • No hope • You want to be all by yourself	Describes the feeling using out of scope phrases or synonyms	• Feel like hurting yourself • Constantly worrying • Not being able to cope
‘Been concerned/ troubled about having little interest or pleasure in doing things’	Describes the feeling using other phrases or synonyms	• It is an effort to do anything • Want to be alone • Feeling pressed down	Describes the feeling using out of scope phrases or synonyms	• Lazy • Tired • Fatigue
‘had thoughts and plans to harm yourself or commit suicide’	Describes the feeling using other phrases or synonyms	• Take your own life • Hurt yourself • Kill yourself • Harm your baby	Describes the feeling using out of scope phrases or synonyms or had thoughts but had not made plans	No out of scope interpretations

Textual data were quantified or coded numerically, by the lead author, and captured in a spreadsheet for analysis. The process of quantifying textual data helped to counteract bias and improve reliability [31].

Quantitative data were captured in Microsoft Excel and exported to STATA/SE statistical software package version 14.2 (StataCorp., College Station, TX, USA) for analysis. Continuous variables that were not normally distributed were described using medians and interquartile ranges, and associations measured using the Kruskal Wallis test for nonparametric variables. Categorical variables were described using frequency and percentages, and associations measured using Fisher’s exact chi-square test as the sample sizes were small.

ROC curves were used to describe the performance of the 4-item screening tool using the EPDS as the reference standard, for phase 1 and phase 2 separately. Sample size calculations (type I error = 0.05; power = 0.08; AUC = 0.8–0.9) indicated that 10–20 participants were needed. The AUC was used to assess the diagnostic performance of the screening tool. In addition, sensitivity, specificity and the percentage correctly classified were calculated for all cut-points.

## Results

These results report on the understanding of the constructs making up the screening tool, and the ability of the two iterations of the screening tool to correctly identify symptoms of mental illness when compared to the EPDS.

### Understanding the screening tool constructs

The proportion of affirmative answers to the 4-item screening tool questions in the three languages was not significantly different (*p* > 0.05), except for the first question which related to anxiety symptoms in phase 1 (Table 4). In phase 1, significantly more women who spoke Afrikaans as a first language endorsed this item (*p* = 0.047), compared to women who spoke English or isiXhosa as a first language.

**Table 4:** Affirmative (yes) answers to screening tool by phase and language of interview

	English n (%)	Afrikaans n (%)	Xhosa n (%)	*P*-value***
Phase 1^a^ (*n* = 30)	*n* = 14	*n* = 9	*n* = 7	
1. Felt unable to stop worrying, or thinking too much?	5 (35.7)	8 (88.9)	4 (57.1)	0.047
2. Felt down, depressed or hopeless?	3 (21.4)	3 (33.3)	4 (57.1)	0.324
3. Felt little interest or pleasure in doing things that you used to enjoy before?	8 (57.1)	5 (55.6)	6 (85.7)	0.494
4. Had thoughts and plans to harm yourself or commit suicide?	0	1 (11.1)	2 (28.6)	0.076
Phase 2^b^ (*n* = 36)	*n* = 15	*n* = 10	*n* = 10	
1. Felt unable to stop worrying, or thinking too much?	5 (33.3)	6 (60.0)	4 (40.0)	0.471
2. Felt down, depressed or hopeless?	5 (33.3)	5 (50.0)	6 (60.0)	0.400
3. Been concerned/troubled about having little interest or pleasure in doing things?	6 (40.0)	4 (40.0)	6 (60.0)	0.685
4. Had thoughts and plans to harm yourself or commit suicide?	0	1 (10.0)	0	0.571

^a^based on a 4 week recall period, and asks about ‘have you often’

^b^based on a 2 week recall period, and asks about ‘have you on some or most days’

***Fisher’s exact test

The 4-week recall period used in phase 1 was often misinterpreted (41% referred to the correct time period) (Table 5). When using the 4-week recall period, we found that more than half the women interviewed used the time of their learning of their being pregnant as a point of reference. When asked about the time period they had been considering when responding to the screening tool, many women replied ‘*just before I found out I was pregnant*’ or ‘*since I found out I was pregnant*’. After we changed the recall period to 2 weeks (i.e. phase 2), 82% of women understood the time construct to refer to the prior 2 week period. When asked about the time period they were thinking about, many women referred to ‘*this week and last week*’, or ‘*in the last two weeks*’, or ‘*two weeks ago*’. When asked about the frequency of the symptoms, ‘often’ was interpreted by many to refer to ‘*now and then*’, ‘*once*’, ‘*twice*’ or they referred to a specific day or event. Hence, the language of the frequency construct was changed to ‘*on some or most days*’ in phase 2. This improved the ‘in scope’ interpretation.

**Table 5:** In scope understanding of time construct and questions in the screening tool by phase and language of interview

Phase 1 (based on a 4 week recall period, and asks about ‘have you often’)	English n (%)	Afrikaans n (%)	Xhosa n (%)	Total n (%)
	*n* = 14	*n* = 8	*n* = 7	*n* = 29
1. 4-week time construct	7 (50.0)	1 (12.5)	4 (57.1)	12 (41.4)
2. Felt unable to stop worrying, or thinking too much?	13 (92.8)	6 (75.0)	7 (100)	26 (89.7)
3. Felt down, depressed or hopeless?	12 (78.6)	5 (62.5)	6 (85.7)	23 (79.3)
4. Felt little interest or pleasure in doing things that you used to enjoy before?	7 (50.0)	4 (50.0)	4 (57.1)	15 (51.7)
5. Had thoughts and plans to harm yourself or commit suicide?	14 (100)	7 (87.5)	7 (100)	28 (96.6)
Phase 2 (based on a 2 week recall period, and asks about ‘have you on some or most days’)	*n* = 14	*n* = 10	*n* = 10	*n* = 34
1. 2-week time construct	11 (78.6)	7 (70.0)	10 (100)	28 (82.3)
2. Felt unable to stop worrying, or thinking too much?	14 (100)	8 (80.0)	8 (80.0)	30 (88.2)
3. Felt down, depressed or hopeless?	14 (100)	9 (90.0)	9 (90.0)	32 (94.1)
4. Been concerned/troubled about having little interest or pleasure in doing things?	6 (42.9)	5 (50.0)	4 (40.0)	14 (44.2)
5. Had thoughts and plans to harm yourself or commit suicide?	13 (92.9)	9 (90.0)	10 (100)	32 (94.1)

The first and second questions about feeling ‘unable to stop worrying, or thinking too much’ and feeling ‘down, depressed or hopeless’ were understood to reflect symptoms of anxiety and depression respectively, in both phases and all three languages. Women frequently used the words ‘*stress*’ or ‘*stressful*’ or ‘*stressing*’ to describe both feelings. The two feelings were often linked together, with women explaining that ‘*being unable to stop thinking and worrying*’ would cause them to feel ‘*down, depressed or hopeless*’.

The third question in the screening tool ‘felt little pleasure or interest in doing things that you used to enjoy before?’ was not understood by 48% of participants in phase 1, to refer to the concept of anhedonia, i.e. the inability to feel and experience pleasure in normally pleasurable activities [32]. After adjusting the question in phase 2, to ‘been concerned/troubled about having little interest or pleasure in doing things?’, only 44% of women interpreted the construct within the scope of anhedonia. When the women were asked to give a reason for feeling ‘little pleasure or interest in doing things’ they reported feeling ‘sleepy’, ‘tired’, ‘lazy’ or having ‘low energy’ since they found out about the pregnancy.

The fourth question in the screening tool on ‘thoughts and plans to harm yourself’ was understood in phase 1, by 90% of the women, to reflect both ideation and planning for suicide. The question did not require any change for the second phase.

### Screening tool ability to detect symptoms of depression, anxiety and suicidality

ROC analysis was performed, using the EPDS as the reference standard (cut-point ≥13), for phase 1 and phase 2 separately (Table 6). The AUC was higher in phase 2 (AUC = 0.959 & 0.928) compared to phase 1 (AUC = 0.841 & 0.865), for both the 4-item and 3-item (without the anhedonia question) screening tools respectively. In both phase 1 and phase 2, when using the same cut-point of ≥2, the 3-item screening tool correctly classified a greater proportion of the sample than the 4-item screening tool. The 3-item screening tool was able to correctly classify 87% in phase 1, and 91% in phase 2, while the 4-item screening tool correctly classified 73% in phase 1 and 74% in phase 2.

**Table 6:** ROC analysis of 4-question screening tool against the EPDS (using ≥13 as the cut-point)

	Score	Phase 1 (*n* = 30)				Phase 2 (*n* = 36)			
		AUC	Sensitivity (%)	Specificity (%)	% correctly classified	AUC	Sensitivity (%)	Specificity (%)	% correctly classified
4-item screening tool	≥1	0.841	88.89	28.57	46.67	0.959	100	35.71	48.57
4-item screening tool	≥2	0.841	88.89	66.67	73.33	0.959	100	67.86	74.29
4-item screening tool	≥3	0.841	66.67	90.48	83.33	0.959	85.71	96.43	94.29
4-item screening tool	4	0.841	33.33	100	80.00	0.959	0	100	80.00
3-item^a^ screening tool	≥1	0.865	88.89	52.38	63.33	0.928	100	42.86	54.29
3-item^a^ screening tool	≥2	0.865	77.78	90.48	86.67	0.928	85.71	92.86	91.43
3-item^a^ screening tool	3	0.865	33.33	100	80.00	0.928	14.29	100	82.86

^a^without the anhedonia question

## Discussion

We used cognitive interviewing to validate the content and structure of a 4-item screening tool used to screen pregnant women for symptoms of CMD and suicidality. Across the three languages, despite some significant differences in socio-demographic characteristics, women’s understanding of the various questions were similar. Although the numbers were small, we found that their language did not influence their interpretation of the questions. We found that the 2-week recall period performed better than the 4-week recall. The anhedonia question that referred to feeling ‘little interest or pleasure in doing things’ was poorly understood in both phases of the study and across all languages, as women associated feeling a decreased interest and pleasure in doing things to be related to tiredness commonly experienced in the first trimester of pregnancy. Using ROC analysis with the EPDS as the reference standard, we showed that a 3-item version of the screening tool (without the anhedonia question) was able to correctly classify 91% of the women screened.

A number of screening questionnaires, including the EPDS [33], Whooley [15], Patient Health Questionnaire 9 (PHQ-9) [34], Kessler-10 [35] and GAD-2 [36] have been used in studies to screen perinatal women for CMD and suicidality. These screening tools have recall periods varying from 7 days (EPDS) to 1 month (Whooley and Kessler-10). In addition, the Expanded MINI-International Neuropsychiatric Interview [37] is a structured diagnostic interview which refers to a 2-week recall period to diagnose current depression, and a 6-month recall period to diagnose a current anxiety disorder as per the latest versions of the major diagnostic manuals used globally: the DSM 5 (Diagnostic and Statistical Manual of Mental Disorders) [38] and the ICD (International Classification of Diseases) [39].

When using the 4-week recall period, we found that more than half the women interviewed did not consider how they had felt before their pregnancy as they often referred to ‘just before I found out I was pregnant’ or ‘since I found out I was pregnant’. After changing the recall period to 2 weeks (as used in diagnostic interviews), we observed considerable increased coherence in their understanding of the time construct. We could find no other studies that reported similar findings, possibly due to the limited practice of reporting the participants’ understanding of recall periods. However, there is the possibility that learning one is pregnant may result in an adjustment response that is not necessarily pathological in the socio-economically deprived context in which this study took place. A brief recall period may thus falsely include those still adjusting to the news of their pregnancy by expressing symptoms of depression and anxiety. Furthermore, the items used to generate the tool were originally selected from a regression analysis against a diagnostic gold standard where diagnostic criteria were strictly observed [18].

We found that the word ‘often’ used in phase 1, as well as ‘on some or most days’ used in phase 2, seemed to cause participants to consider a specific day or event rather than considering a continuous period of time. This is supported by a Kenyan study [40] of HIV positive men and women using the PHQ-9 screening tool. Monahan et al. [40] reported that participants found it confusing to relate the phrase ‘more than half the days’ to the 2-week recall period. Similarly, we found that the women, when asked about their feelings ‘on some or most days’ during the last 2 weeks, did not consider the time period being referred to, but instead referred to a specific day or event. This suggests that time periods are not necessarily useful unless asking about specific events (e.g. taking of medication or visits to the health facility).

In both phases of the study, we found that the anhedonia question elicited ‘out of scope’ interpretations resulting in a number of false positives, irrespective of language or recall period. Many women reported feeling tired, sleepy, or lazy during their pregnancy. They reported having little energy to do the things they normally enjoyed doing. Similar high levels of false positives were reported by Darwin et al. [41] in a study using the Whooley question in a sample of British women in the first trimester of pregnancy. While the phrasing of the question may require adjustment to include a more specific explanation, the inclusion of additional phrases would increase the complexity of the questions, thus reducing the functional utility of the tool. As our objective is to validate a tool that is as brief as possible for busy clinical settings, and for use by a range of provider cadres, it was reassuring to note that removal of the item yielded improved psychometric properties. The ROC analysis showed that 91% of women we correctly classified with the remaining three questions.

In South Africa, the prevalence of perinatal mental disorders is high [8, 9, 42] and has been linked to poverty [43]. Untreated anxiety and depression during the perinatal period has significant, intergenerational effects on the health of mothers and children. However, the perinatal period also provides health care workers with a unique opportunity to identify and treat vulnerable women, as more than 90% of pregnant women access health care facilities during this period [44]. Yet, South African public health facilities do not currently provide routine screening to pregnant women as a result of the overburdened health care system, lack of political will, concern about lack of referral sources and institutional stigma [45]. To screen women attending busy maternity clinics routinely, health care providers require a brief, locally validated tool, which is simple to use, culturally relevant, transdiagnostic (i.e. can identify women with symptoms of depression, anxiety and suicidality), and can be administered by non-specialist care providers. While this study has successfully validated such a screening tool, there remains a number of barriers to integrating screening into routinely provided maternal care. Concerns have been raised regarding the acceptability and benefit of routine screening, limitations of screening tools leading to false positives and negatives, the feasibility of follow-up and access to quality mental health care, as well as the financial cost [46, 47]. However, there is also growing evidence that demonstrates how screening and treating CMD in LMIC improves health outcomes [48].

This study has several strengths. We used a well-recognised scientific methodology, namely cognitive interviewing, to understand the way in which the constructs making up the screening tool performed. This method allowed us to analyse interpretative patterns across groups, as well as the accuracy of the translations. We used an iterative approach which allowed us to refine the tool, before conducted the second set of interviews.

This study also has limitations. Due to limited funds, we did not compare our results to a diagnostic interview, but used another screening tool, the EPDS, instead. While this may be a potential threat to the internal validity, the EPDS has been shown to have good sensitivity and specificity compared to diagnostic interviews in South Africa [49]. In addition, the applicability of our findings may be more generalizable to depression than anxiety since the EPDS anxiety sub-scale was only able to correctly classify 61% of pregnant women.

Additional research is needed to compare the ability of the screening tool to identify symptoms of depression, anxiety or suicidality to that of a diagnostic test, in various settings and stages of pregnancy, including the postpartum period and with adolescent mothers.

## Conclusions

In this study, cognitive interviewing methods were used systematically to test the questionnaire items of an ultra-brief screening tool for perinatal CMD and suicidality. This iterative process enabled testing and refining of the language and constructs in order to ascertain that the tool is well understood and effective at identifying pregnant women with symptoms of CMD. In addition, the tool is valid across the three most commonly spoken languages and cultures in Cape Town.

## Data Availability

The datasets used and/or analysed during the current study are available from the corresponding author on reasonable request.

## Abbreviations

AUC: Area under the curve
CMD: Common mental disorders
DSM: Diagnostic and statistical manual
EPDS: Edinburgh Postnatal Depression Scale
GAD: Generalised Anxiety Scale
HREC: Human Research Ethics Committee
ICD: International Classification of Diseases
LMIC: Low- and Middle-Income Countries
MOU: Midwife Obstetric Unit
PHQ: Patient Health Questionnaire
PMHP: Perinatal Mental Health Project
ROC: Receiver Operating Curve
WHO: World Health Organisation
